# Genotypic Profile of *Streptococcus suis* Serotype 2 and Clinical Features of Infection in Humans, Thailand

**DOI:** 10.3201/eid1705.100754

**Published:** 2011-05

**Authors:** Anusak Kerdsin, Surang Dejsirilert, Parichart Puangpatra, Saowalak Sripakdee, Koranan Chumla, Nitsara Boonkerd, Pitimol Polwichai, Susumu Tanimura, Dan Takeuchi, Tatsuya Nakayama, Shota Nakamura, Yukihiro Akeda, Marcelo Gottschalk, Pathom Sawanpanyalert, Kazunori Oishi

**Affiliations:** Author affiliations: Ministry of Public Health, Nonthaburi, Thailand (A. Kerdsin, S. Dejsirilert, P. Puangpatra, S. Sripakdee, K. Chumla, N. Boonkerd, P. Polwichai, P. Sawanpanyalert);; Naresuan University Phayao Campus, Phayao, Thailand (N. Boonkerd);; Hyogo College of Medicine, Nishinomiya, Japan (S. Tanimura);; Osaka University, Osaka, Japan (D. Takeuchi, T. Nakayama, Y. Akeda, K. Oishi);; Thailand-Japan Research Collaboration Center for Emerging and Re-emerging Infections, Nonthaburi (S. Nakamura);; University of Montreal, Quebec, Canada (M. Gottschalk)

**Keywords:** Streptococcus suis, serotype 2, meningitis, sepsis, sequence typing, bacteria, research

## Abstract

To examine associations between clinical features of *Streptococcus suis* serotype 2 infections in humans in Thailand and genotypic profiles of isolates, we conducted a retrospective study during 2006–2008. Of 165 patients for whom bacterial cultures of blood, cerebrospinal fluid, or both were positive for *S. suis* serotype 2, the major multilocus sequence types (STs) found were ST1 (62.4%) and ST104 (25.5%); the latter is unique to Thailand. Clinical features were examined for 158 patients. Infections were sporadic; case-fatality rate for adults was 9.5%, primarily in northern Thailand. Disease incidence peaked during the rainy season. Disease was classified as meningitis (58.9%) or nonmeningitis (41.1%, and included sepsis [35.4%] and others [5.7%]). Although ST1 strains were significantly associated with the meningitis category (p<0.0001), ST104 strains were significantly associated with the nonmeningitis category (p<0.0001). The ST1 and ST104 strains are capable of causing sepsis, but only the ST1 strains commonly cause meningitis.

*Streptococcus suis*, an emerging zoonotic pathogen, causes invasive infections in persons who are in close contact with infected pigs or contaminated pork-derived products (*1*). On the basis of capsular polysaccharides, 33 serotypes of *S. suis* have now been identified. Of these, serotype 2 is the most prevalent type in humans infected with this pathogen (*1*,*2*). Since the largest outbreak of human *S. suis* infection in 2005, in Sichuan Province, People’s Republic of China (*3*), this disease has been increasingly recognized worldwide. The numbers of reported cases, especially in persons from Southeast Asian countries, have increased dramatically during past few years (*4*).

In Thailand, at least 300 cases of *S. suis* infection in humans have been reported (*5*–*11*). Although an outbreak of *S. suis* infections was confirmed in Phayao Province during May 2007 (*9*), most cases in humans occur sporadically and are primarily located in the northern region of this country (*6*–*11*). A relatively low incidence of cases with *S. suis* serotype 14 has also been reported in this region (*12*). Although previous studies have reported high frequencies (59.0%–88.7%) of *S. suis* infections in persons in this area who ate raw pork products (*8*–*11*), the pathogenesis of this disease, including routes of transmission, is unclear.

The major clinical manifestations of the disease are bacterial meningitis and sepsis, but other manifestations have been reported (*1*,*4*,*8*,*10*,*13*). Most cases of bacterial meningitis can be attributed to the hematogenic spread of invasive bacteria, but how circulating bacteria cross the blood–cerebrospinal fluid (CSF) barrier and cause meningitis is not clear (*14*,*15*). Furthermore, the overall clinical features of this disease have not been extensively and comprehensively investigated in Southeast Asian countries.

A variety of virulence factors associated with *S. suis* have been reported (*16*–*20*), but none have been proven to be essential for the host defense of this disease, except the capsular polysaccharide (*19*). In serotype 2 isolates obtained during a previous outbreak in Sichuan, China, an ≈89-kb DNA fragment, which has been associated with a pathogenicity island (89K PAI), was identified (*21*). The 89K PAI fragment encodes a 2-compartment signal transduction system, SalK-SalR, which is required for full virulence (*22*).

We report the results of a retrospective study of the clinical features of 158 cases of human infection with *S. suis* serotype 2 and the molecular epidemiology of 165 *S. suis* serotype 2 isolates. The study objective was to demonstrate associations between the clinical features of disease caused by *S. suis* serotype 2 in persons in Thailand and the genotypic profiles of the isolates. The study was reviewed and approved by the Ethics Committees of Research Institute for Microbial Diseases, Osaka University, and conducted according to the principles expressed in the Declaration of Helsinki.

## Methods

### Isolate Identification

From January 2006 through August 2008, a total of 1,154 unidentified streptococcal isolates from blood or CSF were collected from hospitals in all 76 provinces of Thailand. Biochemical testing of these isolates, using API Strep (bioMérieux, Durham, NC, USA) and *S. suis*–specific and *S. suis* serotype 2– or 1/2–specific PCR (*12*,*23*), confirmed 165 isolates from 34 hospitals in 25 provinces as *S. suis*. The final serotype of all strains was confirmed by coagglutination tests that used rabbit antiserum (Statens Serum Institute, Copenhagen, Denmark).

### Genotypic Profiles of Isolates

Multilocus sequence type (MLST) testing was performed as described by King et al. (*24*), with a modification for *mutS* as described by Rehm et al (*25*). MLST alleles and the resulting sequence type (ST) were assigned by using the *S. suis* MLST database (http://ssuis.mlst.net). eBURST was used to identify the clonal complexes for these 165 serotype 2 strains within *S. suis*, and the overall structure of the population was obtained through the MLST database (*26*). Virulence-associated genes (VAG), including extracellular released protein factor (*epf*), muramidase-released protein (*mrp*), and suilysin (*sly*), and variants of *mrp* or *epf* were determined by PCR as described by Silva et al. (27), with minor modifications. Presence of the 89K PAI fragment was determined by PCR as reported by Chen et al (*27*). Pulsed-field gel electrophoresis (PFGE) was performed as described (*28*), and the pulsotypes were assigned to clusters of isolates with >80% similarity from the dendrogram. The dendrogram representing the genetic relationships between the representative pulsotypes from 165 *S. suis* serotype 2 strains was drawn by using the Cluster 3.0 software program and examined by using the TreeView program as described (*12*,*29*).

### Clinical Features of Cases

Of the 165 patients whose culture results were positive for *S. suis* serotype 2, medical records for 158 were retrospectively reviewed by physicians at local hospitals in Thailand. Medical records for the remaining 7 patients were not available. The clinical manifestations were mostly divided into 2 categories: meningitis and nonmeningitis. The meningitis category involved confirmed meningitis, bacteremic meningitis, and probable meningitis. All patients in the meningitis category had typical meningeal signs, such as neck stiffness, and acute disease onset. Although bacteremic meningitis was defined as a case in which both CSF and blood cultures were positive, confirmed meningitis was defined as a case with a positive CSF culture only, and probable meningitis was defined as a case with a positive blood culture only. The nonmeningitis category included the clinical manifestations of sepsis and sepsis with focal signs other than meningitis (septic arthritis or spondylodiscitis, infective endocarditis, and bacteremic pneumonia). Sepsis was defined as systemic inflammatory response syndrome and a positive blood culture (*30*), and septic arthritis or septic spondylodiscitis was defined as described (*31*). Diagnosis of infectious endocarditis was based on the Duke criteria (*32*). Septic shock was also defined as described (*33*).

### Statistical Analyses

Comparisons of the clinical characteristics between fatal and nonfatal cases were analyzed by using the χ^2^ test or Fisher exact test with Stata version 10.0 software (StataCorp, College Station, TX, USA). Patient ages and periods of hospital admission were tested for normality of the distribution using the Kolmogorov-Smirnov test and were compared by using the Student *t* test with SPSS version 11.0 software (SPSS Inc., Chicago, IL, USA). Data were considered significant at p<0.05.

## Results

### Genotypic Profiles of Isolates

Of the 165 *S. suis* serotype 2 isolates, 123 were isolated from blood and 42 from CSF. eBURST analysis based on MLST enabled classification of these strains into 4 ST complexes: the ST1, ST27, ST29, and ST104 complexes (Table 1). ST126, a novel ST, has a single locus variant from ST1. The largest cluster of 89K PAI–carrying strains was ST1 (n = 81, 49.1%), which had the *epf*+/*sly*+/*mrp*+ genotype; these strains were isolated from blood and CSF. Another large cluster of non-89K PAI–carrying strains was ST104, which had the *epf–*/*sly*+/*mrp–* genotype (n = 39, 23.6%); most of these strains (n = 38) were isolated only from blood. ST103, ST104, and ST126 were found only in isolates from humans in Thailand.

**Table 1:** Genotypic profiles of 165 clinical isolates of *Streptococcus suis* serotype 2, Thailand, January 2006–August 2008*

ST complex	ST	VAG†	Isolation site	89K PAI		No. (%) strains
				+	–	
1	1	*epf–/sly*+/*mrp*+	Blood	1	0	103 (62.4)
		*epf*+/*sly*+/*mrp*+	Blood	52	13	
			CSF	29	5	
		*epf*+/*sly*+/*mrp*^s^	Blood	0	1	
			CSF	0	2	
	126	*epf*+/*sly*+/*mrp*+	Blood	1	0	3 (1.8)
			CSF	2	0	
27	28	*epf*–/*sly*–/*mrp*+	Blood	0	1	3 (1.8)
			CSF	0	2	
29	25	*epf–/sly*–/mrp*	Blood	8	0	11 (6.7)
		*epf–*/*sly*–/*mrp*+	Blood	3	0	
	103	*epf–/sly*–/mrp*	Blood	2	0	3 (1.8)
		*epf–/sly*–/*mrp*+	Blood	1	0	
104	104	*epf–/sly*+/*mrp*–	Blood	3	38	42 (25.5)
			CSF	0	1	
Total no. strains	–	–	–	102	63	165 (100)

*ST, sequence type; VAG, virulence-associated gene; 89K PAI, an ≈89-kb pathogenicity island; CSF, cerebrospinal fluid; –, not applicable. †*mrp*^s^ and mrp* are *mrp* variants that produce ≈750-bp and ≈1,800-bp fragments, respectively, by PCR (*23*,*34*).

### PFGE of Isolates

**Figure 1:**
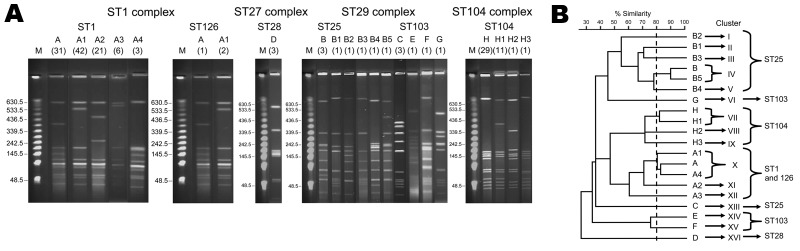
A) Pulsed-field gel electrophoresis profiles of 165 human isolates of *Streptococcus suis* serotype 2, after *Sma*I digestion. Numbers of isolates are indicated in parentheses below pulsotype numbers. B) Dendrogram generated from the pulsed-field gel electrophoresis profiles. ST, sequence type**.**

Of the 165 serotype 2 strains, PFGE analyses identified 20 pulsotypes (Figure 1, panel A). Analysis of the dendrogram for these 20 pulsotypes revealed at least 16 clusters (I to XVI) (Figure 1, panel B). Although 5 pulsotypes of A were identified for the ST1 and ST126 strains, 2 major pulsotypes (A [n = 32] and A1 [n = 43]), A1 (n = 43), and A4 (n = 3) were grouped in 1 cluster. Pulsotype A2 (n = 21), which consisted of ST1 strains lacking the 89K PAI fragment, was classified into a distinguished cluster. PFGE showed diverse DNA patterns for strains ST25 and ST103. ST25 strains were classified into 5 clusters of I, II, III, IV, and VIII. ST103 strains were classified into 3 clusters of VI, XIV, and XV. Three ST28 strains lacking 89K PAI exhibited the unique DNA pattern of pulsotype D; these were classified into cluster XVI. Although 4 pulsotypes (H, H1, H2, and H3) were identified for ST104 strains, 2 major pulsotypes (H [n = 29] and H1 [n = 11]) in ST104 strains were classified into cluster VII. Collectively, clusters X and XI for ST1 and ST126 strains and cluster VII for ST104 strains accounted for the major 3 clusters found for cases in Thailand.

### Geographic and Seasonal Distribution

**Figure 2:**
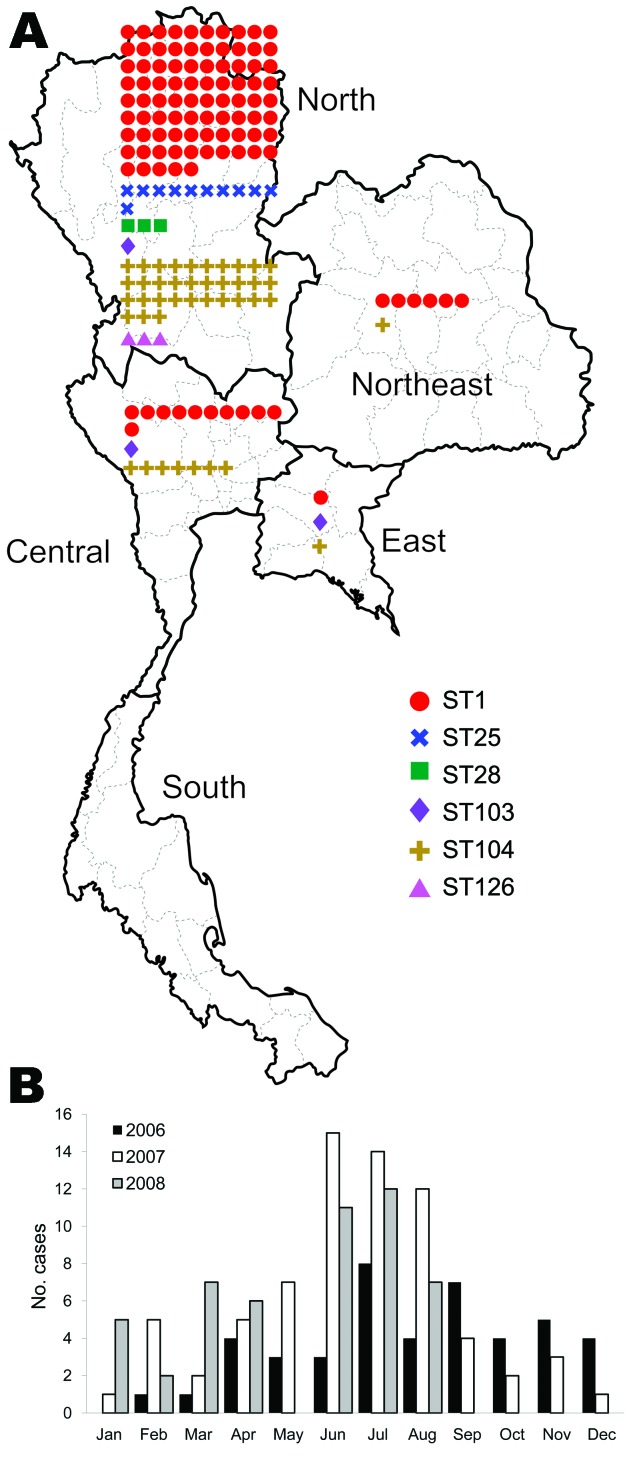
Distribution and sequence types (STs) of 165 human isolates of *Streptocoocus suis* serotype 2, January 2006–August 2008, Thailand. A) Regions of isolation; B) monthly distribution of isolations.

Of the 165 isolates, 136 (82.4%) were from the northern region, 19 (11.5%) from the central region, 7 (4.2%) from the northeast region, and 3 (1.8%) from the eastern region (Table 2; Figure 2, panel A). No strains were isolated from the southern region. The dates of isolation suggest that human cases occur more frequently during the rainy season, June–August of each year (Figure 2, panel B).

**Table 2:** Distribution of sequence types of 165 clinical isolates of *Streptococcus suis* serotype 2, by region, Thailand

Sequence type	North	Northeast	East	Central	South
1	85	6	1	11	0
25	11	0	0	0	0
28	3	0	0	0	0
103	1	0	1	1	0
104	33	1	1	7	0
126	3	0	0	0	0
Total	136	7	3	19	0

### Clinical Features of Cases

The clinical features of the 158 human cases of *S. suis* serotype 2 infection are summarized in Table 3. The median age (range) of the 155 patients for whom age was known was 55.0 (18–93) years; 72.8% were male. No cases in children were identified in this study. All 158 patients had been hospitalized; median duration (range) of hospitalization for the 158 patients was 11 (1–45) days; 15 (9.5%) patients died. No significant differences were found between the fatal and nonfatal cases with respect to patient age or period of admission.

**Table 3:** Demographic and clinical features of 158 human cases of *Streptococcus suis* serotype 2 infections, Thailand, January 2006–August 2008*

Characteristic	All, n = 158	Fatal, n = 15; 9.5%	Nonfatal, n = 143; 90.5%	p value
Demographic				
Male sex, %	72.8	66.7	73.4	0.386
Mean (median) age, y†	56.6 (55.0)	53.9 (52.5)	57.0 (56.0)	0.264
Period of admission, d, mean (median)	12.5 (11)	10.1 (6)	12.9 (12)	0.737
Meningitis category, no. (%) cases				
Confirmed meningitis	22 (13.9)	1 (6.7)	21 (14.7)	0.348
Bacteremic meningitis	44 (27.8)	1 (6.7)	43 (30.1)‡	0.043
Probable meningitis	27 (17.1)	6 (40.0)	21 (14.7)§	0.013
Nonmeningitis category, no. (%) cases				
Septic arthritis	5 (3.2)	0	5 (3.2)	1
Infective endocarditis	3 (1.9)	1 (6.7)	2 (1.4)	0.905
Bacteremic pneumonia	1 (0.6)	0	1 (0.7)	1
Sepsis	56 (35.4)	6 (40.0)	50 (35.0)	0.698
Signs and symptoms, no. (%) cases				
Diarrhea	28 (17.1)	5 (33.3)	23 (16.1)	0.1
Hearing loss	34 (21.5)	4 (26.7)	30 (21.0)	0.409
Altered consciousness	35 (22.2)	4 (26.7)	31 (21.7)	0.434
Shock	9 (5.7)	2 (13.3)	7 (4.9)	0.205
Possible risk factors, no. (%) cases				
Recent consumption of raw pork products	52 (32.9)	5 (33.3)	47 (32.9)	0.589
Recent exposure to pigs	11 (7.0)	2 (13.3)	9 (6.3)	0.28
Alcohol abuse	33 (21.0)	5 (33.3)	28 (19.6)	0.178

*Statistical analyses were performed by using the χ^2^ or Fisher exact test. †Ages were not available for 3 patients. ‡One case of bacteremic meningitis was associated with pneumonia. §Two cases of probable meningitis were associated with spondylodiscitis.

The meningitis category (n = 93) included 22 cases of confirmed meningitis, 44 cases of bacteremic meningitis, and 27 cases of probable meningitis. The nonmeningitis category (n = 65) included sepsis with focal signs other than meningitis (n = 9) and sepsis (n = 56). Sepsis with focal signs other than meningitis included septic arthritis (n = 5), infective endocarditis (n = 3), and bacteremic pneumonia (n = 1). Of the 15 fatal cases, 8 were assigned to the meningitis category (probable meningitis [n = 6], meningitis [n = 1], bacteremic meningitis [n = 1]), 6 cases were sepsis, and 1 case was infective endocarditis (Table 3). Although the cases of bacteremic meningitis were significantly associated with a nonfatal outcome (p = 0.043), the probable meningitis cases were significantly associated with a fatal outcome (p = 0.013). The combined frequencies for the recent consumption of raw pork products and exposure to pigs were 39.9%. None of the clinical signs or possible risk factors, including recent exposure to pigs or raw pork products, or alcohol abuse, was significantly associated with a fatal outcome. Of the 158 patients, 154 parenterally received antimicrobial drugs, such as ceftriaxone, and data concerning antimicrobial drug treatment were not available for 4. Corticosteroids, such as dexamethasone, were used for only 4 patients.

### Clinical Features and Genotype Profiles

The distributions of STs for the 158 human isolates for the meningitis and nonmeningitis categories are shown in Table 4. Although the ST1 strains were significantly associated with the meningitis category (p<0.0001), the ST104 strains were significantly associated with the nonmeningitis category (p<0.0001). The VAG profile of *epf+/sly+/mrp+*, which was dominant in the ST1 strains, was also significantly associated with the meningitis category (p<0.0001). The VAG profile of *epf–/sly+/mrp–*, which was observed only in the ST104 strains, was also significantly associated with the nonmeningitis category (p<0.0001). Because the largest cluster of 89K PAI–carrying strains was associated with the VAG profile of *epf+/sly+/mrp+*, the presence of 89K PAI was also significantly associated with the meningitis category (p<0.0001). None of the genotypic profiles that included STs, VAG, and presence of 89K PAI were significantly associated with fatal or nonfatal outcomes (data not shown).

**Table 4:** Genotypic features of *Streptococcus suis* serotype 2 as risk factor for meningitis*

Feature	Clinical category, no. (%) strains			p value
	All, n = 158	Meningitis, n = 93	Nonmeningitis, n = 65	
Sequence type				
1	98 (62.0)	73 (78.5)	25 (38.5)	<0.0001†
104	40 (25.3)	6 (6.5)	34 (52.3)	<0.0001‡
25	11 (7.0)	7 (7.5)	4 (6.2)	0.478
28	3 (1.9)	2 (2.2)	1 (1.5)	0.632
103	3 (1.9)	2 (2.2)	1 (1.5)	0.655
126	3 (1.9)	2 (2.2)	0	0.201
VAG profile				
* epf+/sly+/mrp+*	97 (61.4)	72 (79.6)	25 (35.4)	<0.0001†
* epf+/sly+/mrp^s^*	3 (25.3)	3 (3.2)	0 (0)	0.201
* epf–/sly+/mrp–*	40 (25.3)	6 (6.5)	34 (52.3)	<0.0001‡
epf– /sly–/mrp*	10 (6.3)	6 (6.5)	4(6.2)	0.607
* epf–/sly–/mrp+*	7 (4.4)	5 (5.3)	2 (3.1)	0.392
* epf–/sly+/mrp+*	1 (1.0)	1 (1.1)	0 (0)	1
89K PAI profile, 89K PAI+	98 (62.0)	70 (75.3)	28 (43.1)	<0.0001†

*Statistical analyses were performed by using the χ^2^ or Fisher exact test. VAG, virulence-associated gene; 89K PAI, ≈89-kb pathogenicity island. †Significant association with the meningitis category. ‡Significant association with the nonmeningitis category.

## Discussion

Our finding that isolated *S. suis* serotype 2 strains peaked during the rainy season of 2006–2008 confirmed conclusions reached in previous small-scale studies conducted in northern Vietnam and Hong Kong (*35*,*36*). The predominant distribution of these isolates in northern Thailand is also in accordance with previous reports (*6*–*11*). However, why no human cases were identified in southern Thailand remains uncertain. A recent study from Hong Kong reported heavy contamination of *S. suis* in raw pork meat at local supermarkets or wet markets; therefore, a hot and humid climate may facilitate the growth of *S. suis* in raw pork products in those markets (*37*) and increase the risk for *S. suis* infections in humans in northern Thailand. The finding of no cases in children suggests that the routes of transmission are associated with adult behavior.

A recent study from northern Thailand, based on 20 human isolates collected during 1998–2002, reported that the most common isolates of *S. suis* serotype 2 were ST25 (40%), followed by ST1 (15%) and ST103 (15%) (*34*). By contrast, the MLST and PFGE results in this study clearly demonstrated that ST1 strains with major pulsotypes of A, A1 and A2, and ST104 with major pulsotypes of H and H1 were currently circulating in the same region of Thailand during 2006–2008. Collectively, these data suggest dynamic replacement of STs from ST25 to ST1 and ST104 among serotype 2 strains during recent years in this region.

Although *S. suis* serotype 2 has been reported to be the most frequent cause of bacterial meningitis in adults in Vietnam (*13*,*35*), other clinical manifestations, such as sepsis and infectious endocarditis, have also been found to be common in Thailand (*6*,*8*,*11*). Of the 158 human cases in the study reported here, ≈60% were assigned to the meningitis category and ≈35% were sepsis. Other clinical manifestations, including infective endocarditis, were rare. The findings reported here demonstrate significant associations between the ST1 strains and the meningitis category and between the ST104 strains and the nonmeningitis category. These findings indicate that both the ST1 and ST104 strains cause bacteremia and sepsis but that the ST1 strains are more likely to cross the blood–CSF barrier and subsequently result in meningitis. Because ≈80% of the cases in the meningitis category were caused by strains with ST1, as evidenced by a VAG profile of *epf*+/*sly*+/*mrp*+ and 89K PAI, these genotypic profiles of *S. suis* serotype 2 may favor bacterial survival and multiplication in the bloodstream, which would result in high levels of bacteremia, crossing of the blood–CSF barrier, and invasion of the meninges and the central nervous system (*15*). Our PFGE data showed that the pulsotype A1 found in serotype 2 strains with ST1 was identical to pulsotype 11 of serotype 2 strains with ST1 from Vietnam and pulsotype I of the serotype 2 strains with ST1 from Hong Kong (*13*,*28*). These isolates from Vietnam and Hong Kong were associated with a VAG profile of *epf*+/*sly*+/*mrp*+, and the strains from Vietnam were also the cause of meningitis in adults. A unique DNA pattern of pulsotype D, classified into cluster XVI, was found for 3 strains with ST28 isolated from nonfatal cases in this study. Previous studies also reported 1 nonfatal case caused by the ST28 strain from Thailand and Japan (*34*,*38*).

Associations for bacteremic meningitis cases with nonfatal outcomes and probable meningitis cases with fatal outcomes contrasted strikingly in this study. Of 6 fatal cases of probable meningitis, 2 were caused by ST1, 2 by ST25, and 2 by ST104 strains. The extent to which the virulence of each ST strain contributed to these deaths remains uncertain. Another possible explanation may be a frequent involvement of critically ill patients, for whom lumbar puncture was not possible; these patients had probable meningitis and typical meningeal signs, acute disease onset, and positive blood culture only.

Because the clinical charts were retrospectively reviewed and the etiologic diagnosis of *S. suis* infection might not have been readily reported to the attending physicians during the hospitalization of the patients in this study, the extent of investigations of clinical manifestations, possible risk factors, and causes of death might have been limited. Because different physicians were involved in the assessment of different patients in this study, the possibility of misdiagnosis for clinical categories cannot be completely excluded even though meningeal signs and acute disease onset are clinical indicators of meningitis.

In conclusion, this study of the clinical features of 158 cases of *S. suis* seotype 2 infection in humans in Thailand showed that the disease occurs sporadically in adults and results in a mortality rate of ≈9.5%; the major clinical manifestations include meningitis and sepsis. MLST analyses of 165 isolates from humans indicated that the major STs were ST1 followed by ST104. Although both ST1 and ST104 strains cause sepsis, it is likely that only the ST1 strain causes meningitis. Further studies are needed to elucidate the pathogenesis of the human *S. suis* infections that are prevalent in Southeast Asian countries.
